# The LSD1 inhibitor iadademstat (ORY-1001) targets SOX2-driven breast cancer stem cells: a potential epigenetic therapy in luminal-B and HER2-positive breast cancer subtypes

**DOI:** 10.18632/aging.102887

**Published:** 2020-03-18

**Authors:** Elisabet Cuyàs, Juan Gumuzio, Sara Verdura, Joan Brunet, Joaquim Bosch-Barrera, Begoña Martin-Castillo, Tomás Alarcón, José Antonio Encinar, Ángel G. Martin, Javier A. Menendez

**Affiliations:** 1 Program Against Cancer Therapeutic Resistance (ProCURE), Metabolism and Cancer Group, Catalan Institute of Oncology, Girona, Spain; 2 Girona Biomedical Research Institute (IDIBGI), Girona, Spain; 3 StemTek Therapeutics, Bilbao, Spain; 4 Medical Oncology, Catalan Institute of Oncology (ICO), Girona, Spain; 5 Department of Medical Sciences, Medical School University of Girona, Girona, Spain; 6 Hereditary Cancer Program, Catalan Institute of Oncology (ICO), Bellvitge Institute for Biomedical Research (IDIBELL), L'Hospitalet del Llobregat, Barcelona, Spain; 7 Hereditary Cancer Program, Catalan Institute of Oncology (ICO), Girona Biomedical Research Institute (IDIBGI), Girona, Spain; 8 Unit of Clinical Research, Catalan Institute of Oncology, Girona, Spain; 9 ICREA, Barcelona, Spain; 10 Centre de Recerca Matemàtica (CRM), Barcelona, Spain; 11 Departament de Matemàtiques, Universitat Autònoma de Barcelona, Barcelona, Spain; 12 Barcelona Graduate School of Mathematics (BGSMath), Barcelona, Spain; 13 Institute of Research, Development and Innovation in Biotechnology of Elche (IDiBE) and Molecular and Cell Biology Institute (IBMC), Miguel Hernández University (UMH), Elche, Spain

**Keywords:** epigenetics, reprogramming, cancer stem cells, breast cancer, patient-derived xenografts

## Abstract

SOX2 is a core pluripotency-associated transcription factor causally related to cancer initiation, aggressiveness, and drug resistance by driving the self-renewal and seeding capacity of cancer stem cells (CSC). Here, we tested the ability of the clinically proven inhibitor of the lysine-specific demethylase 1 (LSD1/KDM1A) iadademstat (ORY-100) to target SOX2-driven CSC in breast cancer. Iadademstat blocked CSC-driven mammosphere formation in breast cancer cell lines that are dependent on SOX2 expression to maintain their CSC phenotype. Iadademstat prevented the activation of an LSD1-targeted stemness-specific SOX2 enhancer in CSC-enriched 3-dimensional spheroids. Using high-throughput transcriptional data available from the METABRIC dataset, high expression of SOX2 was significantly more common in luminal-B and HER2-enriched subtypes according to PAM50 classifier and in IntClust1 (high proliferating luminal-B) and IntClust 5 (luminal-B and HER2-amplified) according to integrative clustering. Iadademstat significantly reduced mammospheres formation by CSC-like cells from a multidrug-resistant luminal-B breast cancer patient-derived xenograft but not of those from a treatment-naïve luminal-A patient. Iadademstat reduced the expression of SOX2 in luminal-B but not in luminal-A mammospheres, likely indicating a selective targeting of SOX2-driven CSC. The therapeutic relevance of targeting SOX2-driven breast CSC suggests the potential clinical use of iadademstat as an epigenetic therapy in luminal-B and HER2-positive subtypes.

## INTRODUCTION

The transcription factor SOX2, a master regulator of embryonic and induced pluripotent stem cells [1–4], is causally related to tumor initiation, aggressiveness, and metastasis likely due to its ability to induce and maintain the stemness of cancer stem cells (CSC) [5, 6]. Given that a key mechanism of drug resistance relates to the incapacity of most standard therapeutics to eradicate the minor subpopulation of CSC with self-renewal and seeding capacity, SOX2 has been suggested as an attractive anti-cancer target to prevent CSC-mediated clinical relapse [7, 8]. Unfortunately, the “undruggable” characteristics of transcription factors such as SOX2 has largely constrained the clinical potential of SOX2-centered therapeutic strategies in major cancer types.

Pre-clinical approaches such as SOX2-targeting siRNAs, shRNAs, or miRNAs offer little therapeutic value because of their poor efficacy and delivery. By the same token, zinc-finger-based artificial transcription factors, which can modify the epi-transcriptional state of endogenous promoters with single locus specificity [9, 10], have been employed to reduce SOX2 mRNA and protein *via* targeting of proximal SOX2 promoters in cultured cancer cells and xenografts [7, 11], but their poor *in vivo* delivery to solid tumor tissue limits their usefulness for stable SOX2 down-regulation in a clinical context. Targeting of SOX2-related upstream/downstream signaling pathways has become a more plausible approach, and pharmacological blockade of either the FBXW2-MSX2 axis with pevonedistat [12], the EGFR-STAT3 pathway with the cationic triphenylmethane pharmacophore gentian violet [13], or EGFR/SRC/AKT signaling with the EGFR inhibitors gefitinib and erlotinib and the Src inhibitor dasatinib [14], have been proposed as strategies to target human cancers with SOX2 overexpression. It is unknown, however, how much of the anti-cancer activity of these indirect approaches can be attributable to SOX2 depletion. Moreover, the aforementioned strategies mostly target the proximal promoters of the *SOX2* gene driving SOX2 expression in the differentiated states of cancer cells, and epigenetic re-activation of stemness-specific enhancers that cause a subpopulation of tumor cells to shift towards a CSC state is unaffected. Mechanistically, such an approach can be achieved by inactivation of lysine-specific demethylase 1 (LSD1/KDM1A), a flavin adenine dinucleotide (FAD)-dependent homolog of the amine oxidase family that demethylates monomethyl or dimethyl lysine 4 (K4) of histone H3. LSD1 blockade with the small molecule inhibitor CBB1007 has been shown to enhance repressive H3K9 methylation at the stemness-specific enhancer of SOX2, thereby validating the notion that LSD1 might serve as a selective epigenetic target for therapeutic ablation of SOX2-driven cancer stemness [15]. Although CBB1007-like competitive LSD1 inhibitors, which have been developed based on the structure of LSD1 with a peptide inhibitor derived from the N-terminal tail of histone H3 [16], might be considered good candidates to selectively target CSC with SOX2-driven pluripotent stem cell properties [17], most of them are in a preclinical stage.

Iadademstat (formerly ORY-1001; Oryzon Genomics, Barcelona, Spain), a clinically proven, highly potent and selective covalent small-molecule inhibitor of LSD1 [18–22], is an emerging therapeutic in hematological malignancies. Iadademstat has been shown to induce blast cell differentiation and reduce the leukemia-propagating stem cell compartment in acute myeloid leukemia (AML). Initial results from a Phase I/IIa clinical trial of iadademstat demonstrated its safety and good tolerability together with preliminary signs of anti-leukemic activity in refractory and relapsed AML [20]. Based on these findings, the Phase IIa ALICE study is currently ongoing in elderly patients with AML not eligible for intensive chemotherapy to combine iadademstat with standard of care azacytidine (https://www.clinicaltrialsregister.eu/ctr-search/trial/2018-000482-36/ES). Beyond hematological cancers, blocking LSD1 with iadademstat has been proposed as a valid strategy in some solid tumors such as small-cell lung cancer (SCLC) and melanoma [21, 22]. Indeed, the Phase II CLEPSIDRA trial is recruiting relapsed SCLC patients to receive iadademstat in combination with platinum-etoposide chemotherapy (https://www.clinicaltrialsregister.eu/ctr-search/trial/2018-000469-35/ES). In addition, the capacity of iadademstat-driven inhibition of LSD1 activity to activate immune responses has recently been proposed as a new means to overcome resistance to immune checkpoint inhibitors in melanoma [22]. Iadademstat-driven reversion of tumor-driving undifferentiated cell states in genomically-diverse malignancies strongly supports the notion that LSD1 might serve as a highly selective epigenetic target for the elimination of cancer cells with pluripotent stem cell-like properties [15, 16, 23, 24]. To test this hypothesis, we here investigated the ability of iadademstat to target SOX2-driven CSC in breast cancer, an unexplored cancer type for iadademstat-based therapy.

Because the mechanism of action of iadademstat has been proposed to either impede the removal of the methyl group from mono-methylated and di-methylated K4 and K9 of histone 3 on LSD1-targeted genes *via* a catalytic/enzymatic mechanism [18], or to promote enhancer activation of subordinate genes through the displacement of LSD1 from chromatin *via* a scaffolding/structural mechanism [19], we first computationally investigated the capacity of iadademstat to target the LSD1-bound FAD cofactor and to disturb the anchorage of LSD1 and its co-repressor (RCOR1/CoREST) to chromatin. Second, because epigenetic re-activation of SOX2 expression via a pluripotency-specific enhancer can cause a subpopulation of tumor cells to dynamically acquire a CSC state, we evaluated the capacity of iadademstat to target the mammosphere-forming capacity -a well-accepted surrogate reporter of CSC activity- in established *in vitro* models bearing distinct mutational landscapes (i.e., BRCA1-mutated basal-like MDA-MB-436 and HER2 gene-amplified/luminal-B BT-474 cell lines) but sharing a common dependency on SOX2 expression to maintain their CSC phenotype. Third, because SOX2 confers sensitivity to LSD1 inhibition, we characterized the SOX2 expression pattern using the PAM50 classifier and the integrative clustering of transcriptional data available from the Molecular Taxonomy of Breast Cancer International consortium (METABRIC). Fourth, we finally evaluated the clinical relevance of iadademstat as a novel anti-SOX2 epigenetic breast cancer therapy by assessing its ability to impact both the expression of SOX2 and the tumorsphere-forming capacity of CSC-like cells derived from breast cancer patient-derived xenografts (PDX).

## RESULTS

### Binding mode of iadademstat to the LSD1-CoREST-histone H3 complex

**Figure 1:**
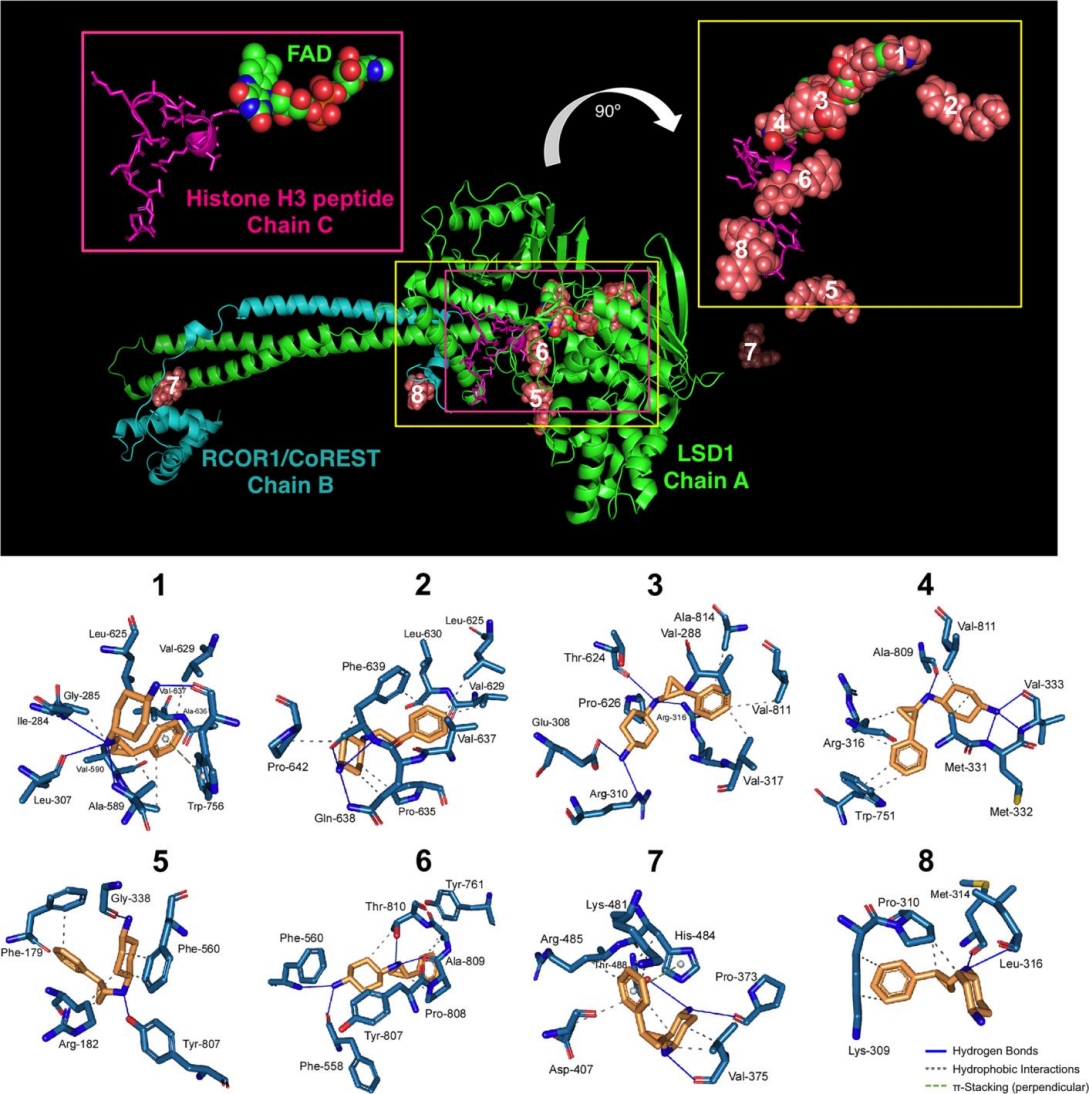
**Binding mode of iadademstat to LSD1.** *Top.* The backbone of LSD1 (chain A)/REST corepressor 1 (chain B)/histone H3 peptide (chain C) heterotrimeric complex is shown. For each cluster of the docked iadademstat (salmon color), only the molecule (spheres) with better binding energy is shown. The molecular docking was performed using the A and B chains in the absence of FAD and histone H3 peptide; however, the clusters of docked iadademstat are shown superimposed on the position that would occupy both the FAD and the histone H3 peptide. The cluster number is also indicated. The insert on the left shows the peptide histone H3 (chain C, backbone as cartoon and side chains as sticks) and the FAD (represented as spheres and with the green carbons). The insert on the right shows only the best pose of iadademstat docked in each cluster and the situation of the histone H3 peptide. The clusters #1, #3 and #4 of iadademstat would occupy the same position of the FAD and are shown superimposed. *Bottom.* The detailed map of the molecular interactions of iadademstat in each cluster is detailed (see also Table 1). Each inset shows the detailed interactions of each compound docked to the LSD1 heterodimer, indicating the participating amino acids involved in the interaction and the type of interaction (hydrogen bonds, hydrophilic interactions, salt bridges, Π-stacking, etc).

Two different models have been proposed to explain the mechanism of action of iadademstat. In the first, iadademstat rapidly and irreversibly binds the LSD1 cofactor FAD in a manner analogous to the monoamine oxidase inhibitor tranylcypromine [18]. In the second, iadademstat physically separates LSD1/RCOR1 from the SNAG-domain transcription repressor GFI1 and chromatin in a cell-type-specific manner [19]. Taking advantage of the solved three-dimensional structure of LSD1 in a ternary complex with its histone peptide substrate and RCOR1/CoREST, we aimed to computationally explore a working model whereby iadademstat might operate *via* both a catalytic/enzymatic mechanism involving highly potent, direct targeting of the FAD cofactor (at lower concentrations), and a scaffolding/structural mechanism involving inhibition of the chromatin binding activity of LSD1/RCOR1 (at higher concentrations). Docking simulations of iadademstat in a crystal structure of human LSD1 (chain A) including RCOR1/CoREST (chain B) – a co-repressor that collaborates to demethylate mono- and di-methylated H3-K9 in nucleosomes – and a histone H3 peptide (chain C), produced eight clusters of docking poses (Figure 1). When the docking results were ranked according to the ascent of the binding energies for iadademstat (up to -9.09 kcal/mol; Table 1), those clusters exhibiting the highest affinity (#1, #3, and #4, see inset on the top right of Figure 1) were in the nanomolar range and were predicted to occupy the same binding site as FAD in LSD1. Cluster #6 was predicted to interfere with the position of the histone H3 peptide, whereas clusters #7 and #8, with affinities in the low micromolar range, were predicted to interact with both the LSD1 enzyme and RCOR1/CoREST.

**Table 1:** Details of the interaction of iadademstat docked to the LSD1 heterodimer (see Figure 1A).

**Cluster number**	**ΔG, [kcal/mol]**	**Dissoc. constant, [μM]**	**Members**	**MM/PBSA solvation binding energy [kcal/mol]**	**Residues of the receptor that contact iadademstat**
1	-9.09	0.21811	5%	-10.018	Ile-284, Gly-285, Ser-286, Gly-287, Leu-307, Glu-308, Ala-309, Arg-310, Gly-315, Thr-588, Ala-589, Val-590, Arg-591, Thr-624, Leu-625, Pro-626, Val-629, Gln-632, Ala-636, Val-637, Trp-756, Ala-757 (**chain A**)
2	-8.84	0.32872	9%	-21.945	Leu-625, Val-629, Leu-630, Lys-631, Gln-632, Gln-633, Pro-635, Ala-636, Val-637, Gln-638, Phe-639, Val-640, Pro-642, Leu-643, Thr-648 (**chain A**)
3	-8.75	0.38632	5.5%	-103.909	Gly-285, Ser-286, Gly-287, Val-288, Ser-289, Gly-290, Glu-308, Ala-309, Arg-310, Gly-314, Gly-315, Arg-316, Val-317, Thr-624, Leu-625, Pro-626, Trp-756, Gly-800, Glu-801, Thr-810, Val-811, Ala-814 (**chain A**)
4	-8.74	0.39379	17%	-111.598	Arg-316, Leu-329, Gly-330, Ala-331, Met-332, Val-333, Thr-335, Tyr-571, Leu-659, Asn-660, Lys-661, Trp-751, Ser-760, Tyr-761, Ala-809, Thr-810, Val-811 (**chain A**)
5	-7.95	1.49000	4.5%	-68.647	Ala-178, Phe-179, Arg-182, Leu-183, Pro-184, His-185, Gly-338, Gly-339, Asp-557, Phe-558, Glu-559, Phe-560, Thr-561, Tyr-807 (**chain A**)
6	-7.92	1.57000	2.5%	-29.621	Thr-335, Ala-539, Asn 540, Trp-552, Asp-555, Phe-558, Glu-559, Phe-560, Tyr-761, Ser-762, Tyr-763, Val-764, Asn 806, Tyr-807, Pro-808, Ala-809, Thr 810, His-812 (**chain A**)
7	-7.09	6.40000	16.5%	-18.198	Lys-481, Ser-482, His-484, Arg-485, Thr-488 (**chain A**) and Leu-372, Pro-373, Glu-374, Val-375, Ile-376, Gln-377, Asp-407, Val-408, Gly-410 (**chain B**)
8	-6.33	22.98000	8%	-32.309	Tyr-391 (**chain A**) and Lys-309, Pro-310, Pro-311, Lys-312, Gly-313, Met-314, Phe-315, Leu-316, Ser-317, Gln-318 (**chain B**)

For the best-docked iadademstat molecule of each cluster, the Gibbs free energy, the dissociation constant, the number of molecules members (as %), and the MM/PBSA solvation binding energy are shown.

To add protein flexibility and provide additional information about different intra- and inter-molecular movements, we performed short molecular dynamics (MD) simulations over the course of 10 ns together with binding free energy calculations under the Molecular Mechanics Poisson-Boltzmann Surface Area (MM-PBSA) approximation. The results highlighted the extremely high affinity of iadademstat at the FAD-targeted clusters #3, and #4, which reached -103.909 and -111.598 kcal/mol, respectively (Table 1).

Using an AlphaScreen™ assay with a biotinylated histone H3 peptide methylated at lysine 4, purified LSD1, and a highly specific antibody that recognizes demethylated substrate, we confirmed the *in vitro* efficacy of iadademstat to dose-dependently suppress the demethylase activity of LSD1, with a mean IC_50_ of 12 nmol/L (Supplementary Figure 1).

### Iadademstat specifically suppresses the mammosphere-formation potential of breast cancer stem cells

**Figure 2:**
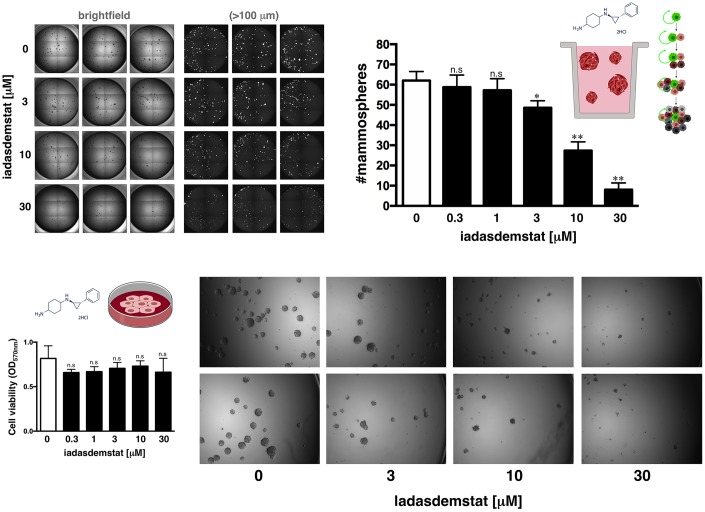
**Iadademstat suppresses mammosphere formation in a basal-like established cell line.** Figure shows representative microscope representations (×2.5 magnification) of mammospheres formed by MDA-MB-436 cells growing in sphere medium for 6 days in the absence or presence of graded concentrations of iadademstat. The number of mammospheres (>100 μm diameter) is expressed as means (*columns*) ± SD (*bars*). MTT uptake-based measurement of cell viability is expressed as percentages uptake (OD_570_) relative to untreated controls (=100% cell viability). The results are expressed as percentages means (*columns*) ± SD (*bars*). *P < 0.05 and **P < 0.005, statistically significant differences from the untreated (control) group.

One of the gold standards for evaluating the presence of CSC is their ability to form *in vitro* mammospheres in low-density non-adherent serum-free medium supplemented with growth factors [25, 26–29]. We assessed the anti-CSC activity of iadademstat in triple-negative breast cancer (TNBC), a highly aggressive breast cancer subtype driven by highly enriched CSC, which are related to therapy resistance, tumor relapse, and metastasis [25, 30]. Accordingly, estrogen receptor (ER)-negative/progesterone receptor (PR)-negative/ HER2-negative BRCA1^mut^/PTEN^mut^ MDA-MB-436 cells, which can form smooth and round spheres in suspension culture [25] and are dependent on SOX2 expression to maintain their CSC phenotype [31], were used in the Cell2Sphere™ assay to evaluate the impact of iadademstat on the ability of CSC to survive and proliferate as floating microtumors. Specifically, we examined the effects of iadademstat on the total number, size, and aspect of MDA-MB-436 mammospheres growing under stem cell-selective conditions. Compared with the untreated controls, exposure to graded concentrations of iadademstat resulted in a dose-dependent decrease in the total number of mammospheres (IC_50_ = 3.98 μmol/L; Figure 2). Notably, the strong decrease in mammosphere formation by iadademstat was not due to non-specific toxicity, as MTT-based cell viability assays run in parallel in 10% serum-supplemented adherent conditions showed no significant cytotoxic activity of iadademstat, even when employing concentrations as high as 30 μmol/L – a dose that completely prevented mammosphere formation in MDA-MB-436 cells (Figure 2).

### Iadademstat suppresses pluripotency enhancer-driven activation of *SOX2* in breast cancer stem cells

**Figure 3:**
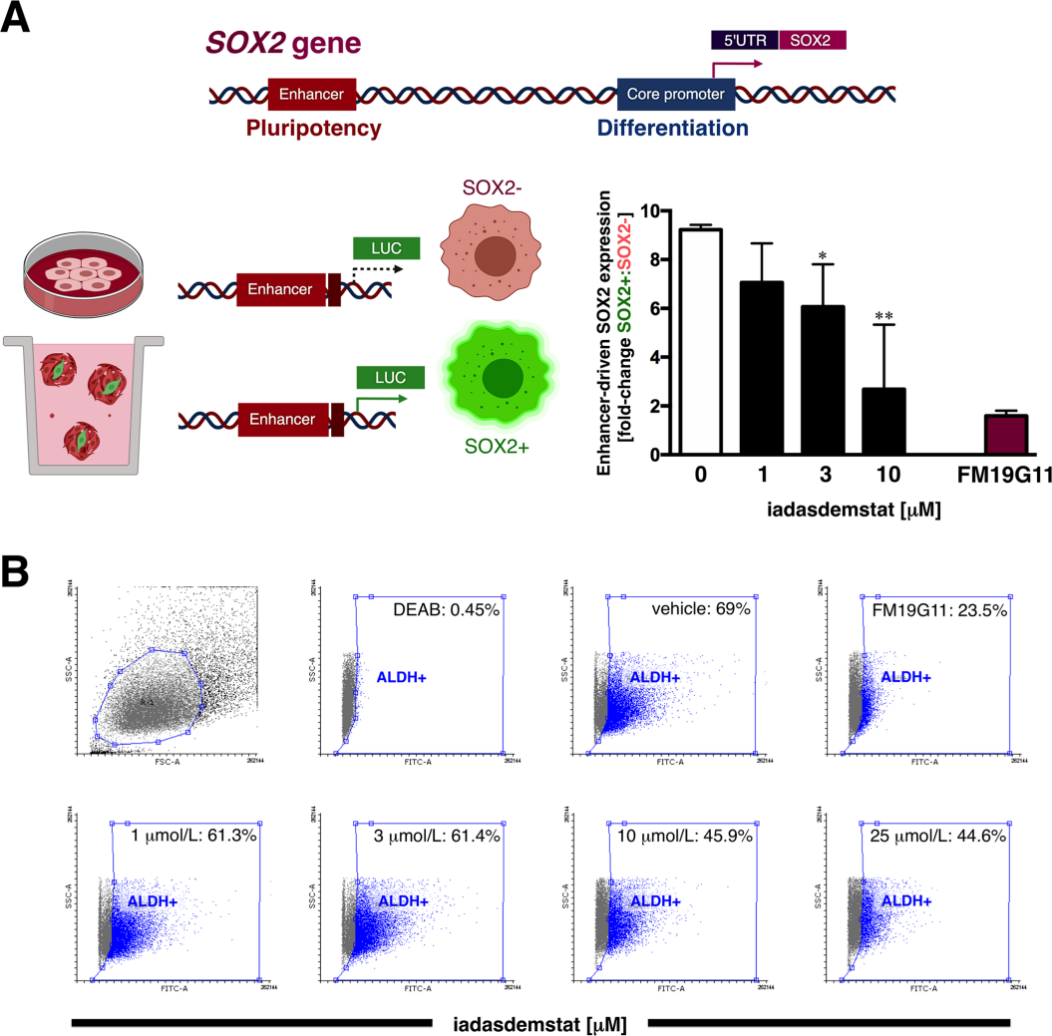
**Iadademstat inhibits stemness-associated *SOX2* expression in a luminal-B/HER2+ established cell line.** (**A**) Schematic representation of *SOX2* promoter structure indicating the proximal core promoter region and the location of the distal enhancer, which is induced exclusively upon CSC-driven mammosphere formation but not in cell-adherent differentiating conditions. Results are expressed as fold-induction of mammosphere culture-associated *SOX2* reporter activity above adherent culture control in the absence or presence of graded concentrations of iadademstat. The results are expressed as percentages means (*columns*) ± SD (*bars*). *P < 0.05 and **P < 0.005, statistically significant differences from the untreated (control) group. (**B**) Representative Aldefluor^®^ assay to identify BT-474 cells with high ALDH activity (ALDH^+^) in the absence or presence of graded concentrations of iadademstat for 3 days. The ALDH inhibitor diethylaminobenzaldehyde (DEAB) was used as negative control. Monolayer cultures were fed with iadademstat on day 1. (Note: 1 μmol/L FM19G11, an epigenetic repressor of key genes involved in stemness including SOX2 [98], was employed as a positive control).

LSD1-blocking compounds are known to differentially target pluripotent cancer cells including teratocarcinoma, embryonic carcinoma, and seminoma, or embryonic stem cells that express *SOX2*, while having minimal growth-inhibitory effects on non-pluripotent cancer or normal somatic cells [15, 16, 23]. We therefore envisioned that iadademstat might suppress CSC function by repressing the re-activation of *SOX2* in breast CSC, a transcriptional phenomenon that specifically occurs through activation of the distal enhancer of the *SOX2* promoter that also controls *SOX2* transcription in pluripotent stem cells [32, 33]. When we transfected tamoxifen-resistant, luminal-B/HER2+ BT-474 cells [34] with a luciferase reporter vector containing the *SOX2* distal enhancer region, we observed a robust induction (9.2-fold on average) of reporter activity in mammosphere cultures when compared with the adherent culture control (Figure 3A). Of note, the enhancer-driven transcriptional activation of *SOX2* differentially occurring in mammosphere cultures was dose-dependently suppressed by iadademstat (up to 80% reduction at 10 μmol/L; Figure 3A). Importantly, iadademstant-driven SOX2 silencing drastically reduced the number of BT-474 mammospheres (data not shown).

### Iadademstat fails to target ALDH+ breast cancer stem cells

We next tested whether the mechanism of action of iadademstat to specifically target mammosphere-forming CSC-like cells might be due to a more general phenomenon involving drivers of breast cancer stemness other than SOX2. Thus, we evaluated its capacity to target cells with high levels of aldehyde dehydrogenase-1 (ALDH1), a biomarker that has been suggested to label a tumorigenic cell fraction capable of self-renewal [35, 36]. ALDH1^+^ cell subpopulations enriched for cancer-initiating activity can be readily identified by flow cytometry using the Aldefluor^®^ reagent, which quantifies ALDH activity by measuring the conversion of the ALDH substrate BODIPY aminoacetaldehyde to the fluorescent product BODIPY aminoacetate. Using HER2-overexpressing BT-474 cells as a breast cancer model naturally enriched with ALDH1^+^ cells (>50%), we detected a decrease in the proportion of ALDH1^+^ cells following 72 h treatment with increasing concentrations of iadademstat (up to 35% decrease at 25 μmol/L iadademstat) as compared with vehicle-treated controls (Figure 3B).

### SOX2 expression status associates with luminal-B and HER2-positive breast carcinomas

**Figure 4:**
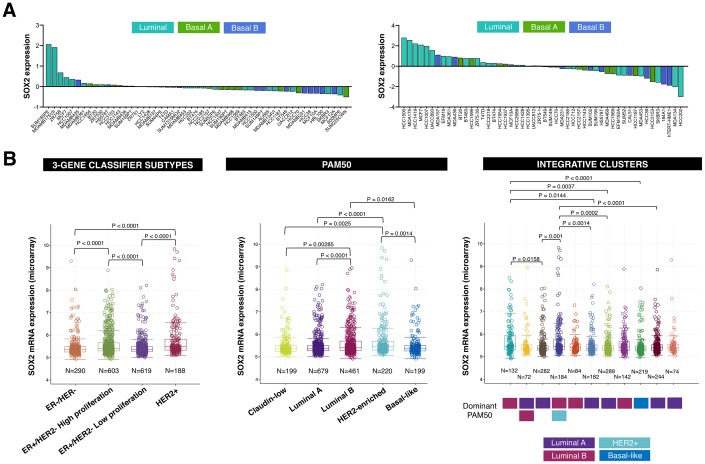
**SOX2 expression is enriched in luminal-B and HER2-positive breast cancer subtypes.** (**A**) Relative enrichment of SOX2 expression (213721_at) in breast cancer cell lines organized by luminal, basal A, and basal B sub-classes [38, 39]. (**B**) Box plots presents the SOX2 gene expression in primary breast tumors from the METABRIC project classified in distinct subtypes using 3-gene (*left*), PAM50 (*middle*), and integrative clusters (*right*) classifiers. The color line presents median, box shows interquartile region and whiskers – the highest (max) and the lowest (min) value.

Comprehensive comparison of molecular portraits between cell lines and breast cancer tumors confirmed the luminal-B intrinsic subtype classification of the ER+/HER2+ BT-474 cell line but revealed that the basal-like cell line MDA-MB-436 exhibits similar protein features to those of the luminal-A breast cancer subtype [37]. Because SOX2-overexpressing cells are particularly sensitive to LSD1 inhibitors [15, 17], we explored the possibility that SOX2 expression might associate with the luminal entity across breast tumor subtypes. When we examined the expression status of SOX2 in two sets of breast cancer cell lines organized by luminal, basal-A (i.e., basal-like breast cancer intrinsic subtype), and basal-B (i.e., claudin-low breast cancer intrinsic subtype) sub-classes [38, 39], most of the SOX2-overexpressing breast carcinoma cell lines were found to belong to the luminal subclass (Figure 4A).

We then extracted breast cancer date sets from the Molecular Taxonomy of Breast Cancer International Consortium (METABRIC) [40, 41] to explore the association between SOX2 expression and multiple breast cancer subtypes. When the METABRIC breast cancer data set was classified into each of the four Gene Expression prognostic Index Using Subtypes (GENIUS) subgroups using a 3-gene classifier (i.e., HER2+, ER-/HER-, ER+/HER2- high proliferation, ER+/HER2- low proliferation) [42, 43], SOX2 expression was found to be significantly higher in the HER2+ and ER+/HER2- high proliferation (luminal B-like) subgroups (Figure 4B, *left panel*). When the METABRIC breast cancer data set was classified into each of the five intrinsic subtypes (i.e., luminal-A, luminal-B, HER2-enriched, basal-like, and normal-like) using the research-based 50-gene prediction analysis of microarray (PAM50) classifier [44], SOX2 expression was found to be significantly higher in the luminal-B and HER2-enriched subtypes (Figure 4B, *middle panel*). When the METABRIC data set was classified into each of the 10 integrative clusters (IntClust1-10) each associated with distinct somatic aberrations (CNAs) and gene expression changes [45, 46], the transcript level of SOX2 was found to be significantly upregulated in the IntClust1, which is constituted by ER-positive tumors predominantly classified into the higher proliferation luminal-B intrinsic subtype, and in the IntClust5, which mostly encompasses HER2-amplified breast composed of both HER2-enriched and luminal-B intrinsic subtypes (Figure 4B, *right panel*).

### Iadademstat suppresses the formation of CSC-enriched mammospheres derived from a multidrug-resistant luminal-B breast cancer patient in a SOX2-related manner

**Figure 5:**
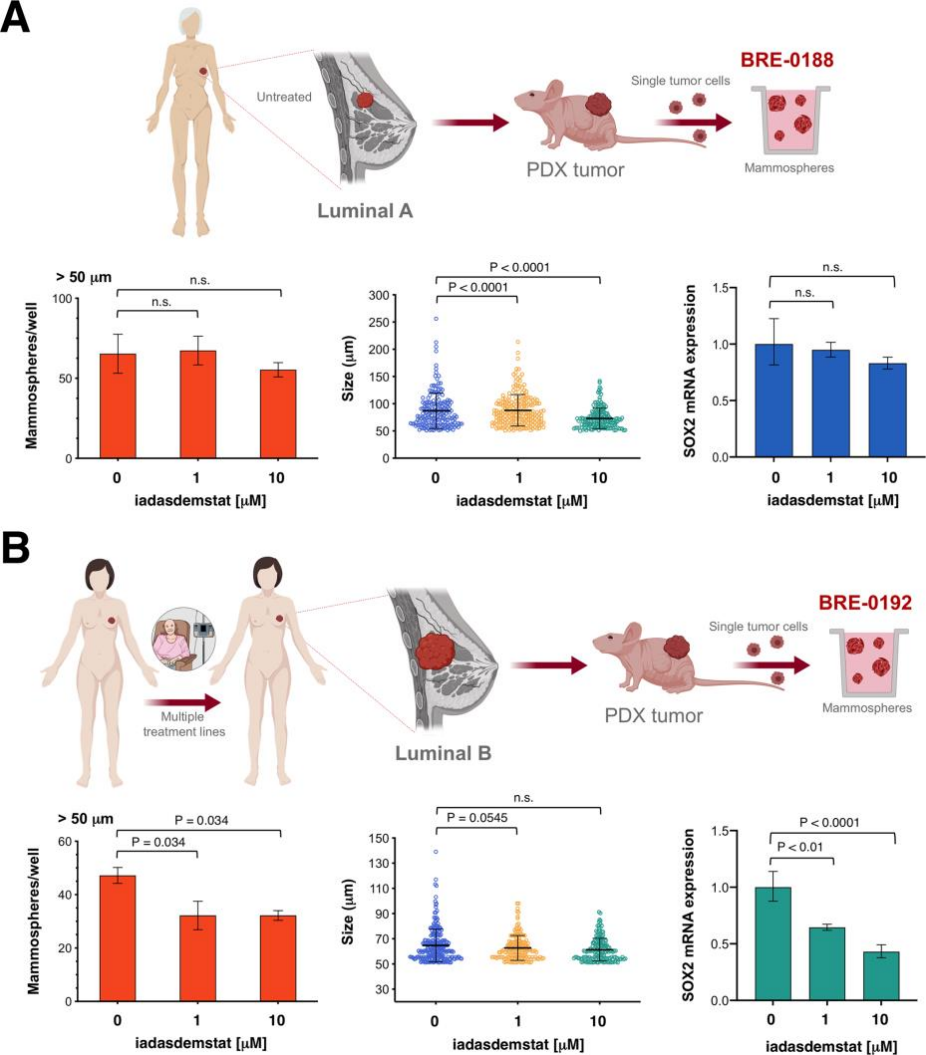
**Iadademstat targets SOX2-driven CSC in breast cancer patient-derived xenografts.** Cell2Sphere™ assays using BRE-0188 (**A**) and BRE-0192 (**B**) PDXs were performed as per the manufacturer’s instructions (http://stemtektherapeutics.com/en/cell2sphere#cell2 sphere_kit). Increasing concentrations of iadademstat were added to sextuplicate sets of wells on day 1. ImageJ was used to quantify the number (*left panels*) and size (*middle panels*; central lines indicate mean values) of 9-day-old mammospheres. *Right panels.* Total RNA from untreated and iadademstat-treated mammosphere cells was evaluated in technical triplicates for the abundance of SOX2 (Hs01053049_s1) relative to housekeeping gene 18S (Hs99999901_s1). The transcript abundance was calculated using the delta Ct method (i.e., the difference of Ct value between the target SOX2 gene and the endogenous 18S control) and presented as relative quantification.

Because patient-derived xenograft (PDX) tumor models more faithful recapitulate human tumor biology and drug responsiveness than established human cancer cell lines [47, 48], we employed CSC-enriched mammospheres generated by growing single-cell suspensions from excised PDX tumors under adherent-free conditions [49] to test the anti-CSC activity of iadademstat in a clinically-relevant scenario (Figure 5). The BRE-0188 (ER+/PR+/HER2-) PDX model was generated from a clinical sample obtained from a 65-year-old female with luminal-A invasive ductal carcinoma with the presence of lymph node metastases. The patients had not received any chemotherapy or radiotherapy prior to surgery. The BRE-0192 (ER+/PR+/HER2-) PDX model was generated from a clinical sample obtained from a 45-year-old female with luminal-B invasive lobular breast carcinoma with the presence of lymph node metastases [50]. The patient was a poor responder to prior therapies, including epirubicin, 5-fluorouracil, cyclophosphamide, taxotere, paclitaxel, bevacizumab, and gemcitabine.

Iadademstat treatment failed to decrease the number of mammospheres originated from CSC-like cells derived from the luminal-A BRE-0188 PDX (Figure 5A); the size of BRE-0188 mammospheres, however, was significantly decreased after treatment with iadademstat. Treatment of BRE-0188 mammospheres cultures with iadademstat failed to alter SOX2 expression. Conversely, iadademstat treatment significantly decreased the number but not the size of mammospheres originated from the multidrug-resistant CSC-like cells derived from the luminal-B BRE-0192 PDX (Figure 5B). Moreover, treatment with iadademstat notably reduced the expression of SOX2 in mammospheres collected from the BRE-0192 PDX.

## DISCUSSION

We provide the first evidence that the clinically proven inhibitor of LSD1 iadademstat can be used to circumvent the challenge of pharmacologically manipulating the epigenetic re-activation of SOX2 that causes a subpopulation of tumor cells to shift towards a CSC state, which has constrained the clinical relevance of SOX2-centered therapeutic strategies in major cancer types such as breast cancer. The therapeutic potential of targeting SOX2-driven CSC supports the clinical use of iadademstat as a novel anti-SOX2 epigenetic breast cancer therapy, particularly in SOX2-enriched luminal-B and HER2-positive subtypes.

Some malignant tumors such as breast carcinoma depend on SOX2 for their tumor-initiating ability [34, 51–53]. Elevated LSD1 levels are associated with enhanced *SOX2* expression, and SOX2-overexpressing cells are particularly sensitive to LSD1 inhibitors. Accordingly, LSD1 has been proposed as a selective epigenetic target for therapy in SOX2-expressing cancers, particularly in those carcinomas in which high SOX2 expression occurs *via* 3q26 chromosomal amplification (e.g., lung SCLC, serous ovarian carcinoma, cervical, head and neck, oral, and esophageal carcinomas [6]). However, such LSD1 inhibitor-based therapeutic strategies might become even more relevant as cancer is entering a new era where the concept of cellular/phenotypic plasticity involving the dynamic interconversion of cells with and without CSC states is challenging how we treat and understand tumors relapse [54–61]. LSD1 controls *SOX2* expression by binding to its distal enhancer, which drives SOX2 activation in pluripotent stem-like cells including CSC [15, 33, 62]. Pharmacological blockade of LSD1 selectively promotes methylation of H3K4 and H3K9 within the regulatory (enhancer) region of the *SOX2* promoter, suppressing SOX2 activity and stimulating cell differentiation by augmenting H3K4 methylation on the promoters of differentiation genes [15]. In a process that is not mutually exclusive, the differentiation genes that are directly controlled by LSD1 can indirectly inhibit the expression of *SOX2* that confers stem cell-like traits to breast cancer cells [63]. Indeed, the therapeutic implications of our current findings with the LSD1 iadademstat might involve molecular scenarios in which the epigenetic re-activation of SOX2 within a group of transformed cells in response to microenvironmental cues, stochastic genetic and epigenetic alterations, and/or treatment-imposed selective pressures can cause them to shift toward a CSC state, LSD1-driven aberrant activation of SOX2 *via* stemness-specific enhancers irrespective of their mutational landscape (e.g., BRCA1-mutated basal-like, HER2 gene-amplified), but also in certain breast cancer intrinsic subtypes that might be intrinsically addicted to the major role of SOX2 in self-renewal growth and expansion of CSC-like cells (i.e., luminal-B and HER2-positive). Such clinically relevant specificity of the LSD1 inhibitor iadademstat against SOX2-driven CSC was confirmed not only in established breast cancer cell lines that are dependent on SOX2 expression to maintain their CSC phenotype [31, 34] but also in CSC-enriched mammospheres generated by growing single-cell suspensions from excised PDX tumors.

The fact that the *SOX2* reporter assay detects variations in *SOX2* transcription regardless of the proportion of cells expressing a CSC marker along with the ability of iadademstat-induced knockdown of SOX2 to sharply decrease the quantity of CSC-like cells capable of self-renewal *in vitro* but not to completely suppress subpopulations with high enzymatic activity of ALDH1 (ALDH1^high^), which has been shown to mark a breast cancer population enriched for proliferating CSC, strongly suggest that the anti-CSC activity of iadademstat might be restricted to SOX2-driven CSC bio-behaviors (e.g., 1-3% of all luminal-B/HER2+ BT-474 cells) that do not necessarily overlap with that of ALDH^high^ proliferating cells. Using CSC-enriched mammospheres obtained by growing single-cell suspensions from excised PDX tumor under adherent-free conditions, treatment with iadademstat resulted in significantly reduced mammosphere formation (but not reduced mammosphere size) in a multi-drug resistant luminal-B breast carcinoma and in significantly reduced mammosphere size (but not reduced mammosphere number) in a treatment-naïve luminal-A breast cancer. The number of mammospheres reflects the quantity of CSC-like cells capable of self-renewal *in vitro*, while the size of mammospheres is an indirect measure of the self-renewal capacity of each mammosphere-generating cell; thus, cell proliferation during mammosphere growth determines the size of the mammospheres [64–66]. Taking together, these results indicate that iadademstant inhibited CSC-driven mammosphere formation efficiency through suppressing LSD1/SOX2 axis in a luminal-B breast carcinoma, whereas the size of mammospheres in a luminal-A tumor was regulated through other underlying proliferative mechanism via LSD1. Accordingly, treatment of mammospheres from the luminal-B (but not the luminal-A) breast cancer patient with iadademstat significantly reduced the expression of SOX2, likely indicating a selective targeting of SOX2-driven CSC. The therapeutic potential of targeting SOX2-driven CSC supports the clinical use of iadademstat as a novel anti-SOX2 epigenetic breast cancer therapy, particularly in endocrine therapy-resistant luminal-B cases -which are known employ SOX2 to increase the proportion of CSC-like cells, rendering them insensitive to tamoxifen [34]- and in HER2-positive disease –in which SOX2 overexpression correlates with poor differentiation [67] and HER2-targeted therapies such as trastuzumab fail to eliminate SOX2-overexpressing CSC [68]. We have to acknowledge that the LSD1/SOX2 axis may represent a provocative potential target for CSC elimination not only in luminal-B and HER2-positive tumor but also in the basal-like subtype, which can also be found in the SOX2-overexpressing IntClust5 [46]. In this regard, because *SOX2* reactivation has been shown to depend on the recruitment of the tumor suppressor protein BRCA1 to the pluripotency-related distal enhancer in the *SOX2* promoter and concomitant modification of H3K4 and H3K9 at the same enhancer [62], our findings in basal-like MDA-MB-436 cells support the idea that BRCA1-mediated predisposition to breast and ovarian cancer [69] might involve a controlled loss of *SOX2* expression, establishing a direct link between LSD1-regulated expression of *SOX2*, acquisition of stem-like cell phenotypes, and BRCA1-related breast/ovarian cancer initiation. Indeed, *SOX2* is frequently gained in *BRCA1* germline mutated tumors and is preferentially expressed in sporadic basal-like phenotypes having similar phenotypic and clinical characteristics to breast cancer arising in *BRCA1* mutation carriers. Accordingly, SOX2 might play a driver role in the development of their less differentiated/stem cell-like phenotypic traits characteristics of the basal-like breast cancer phenotype [70]. Moreover, *SOX2* overexpression occurs not only in high-grade serous ovarian tumors, but also in pre-malignant, fallopian tube epithelial cells from *BRCA1/BRCA2* mutation carriers who underwent prophylactic salpingo-oophorectomy [71] – opening the possibility for considering LSD1-targeted epigenetic approaches such as iadademstat for breast/ovarian cancer prevention.

SOX2 activation has proved instrumental for the plastic acquisition of aberrant stemness properties in cancer cells. Accordingly, certain CSC bio-behaviors can be defined as rare therapy-resistant, self-renewing cancer cells that aberrantly express SOX2, which might provide specificity for CSC-targeted drug screening. The use of fluorescence protein expression-based transcriptional reporters for activation of the LSD1-regulated enhancer element of the *SOX2* gene promoter can specifically identify cells with tumor-initiating activity; compounds that would be capable of impeding SOX2 activation might be viewed as valuable candidates for drugs aimed to target CSC. In this regard, our findings unravel for the first time the ability of iadademstat to inhibit an LSD1-targeted distal enhancer that specifically controls the expression of the stem cell transcription factor SOX2 in pluripotent stem cells, thereby suppressing the re-activation of SOX2 exclusively occurring in mammosphere-initiating breast CSC. The ability of iadademstat to efficiently and specifically target the *on/off* LSD1-driven SOX2 regulatory process that provides higher tumorigenic potential to cells with an epigenetically acquired CSC phenotype [61, 72] might open new therapeutic horizons that incorporate iadademstat in the anti-breast cancer armamentarium. We further propose that iadademstat might functionally deplete tumor-initiating CSC-like cellular states that sustain tumorigenicity by impacting on fundamental controllers of cell fate choice, an epigenetic mechanism involving both the downregulation of *SOX2* and the re-activation of epigenetically suppressed differentiation programs in SOX2-enriched breast cancer subtypes such as Luminal-B and HER2-positive.

Furthermore, we are rapidly appreciating that nuclear reprogramming-like phenomena inducing the acquisition of epigenetic plasticity and phenotype malleability should be viewed as a fundamental element of a tissue’s capacity to undergo successful repair, aging degeneration or malignant transformation [73–77]. Thus, chronic or unrestrained cell plasticity would drive aging phenotypes by impairing the repair or the replacement of damaged cells and such uncontrolled phenomena of *in vivo* reprogramming might also generate CSC-like cellular states [73–77]. Pharmacological tools selectively targeting the LSD1-SOX2 axis might be appropriate to experimentally uncouple the apparently counterintuitive capacity of LSD1 blockade to promote reprogramming phenomena by regulating the balance between pluripotency and differentiation [78–83] while preventing SOX2-driven cancer stemness. This would raise the possibility of pharmacologically managing, in the appropriate direction and intensity, the physiological *versus* pathological processes of SOX2-related reparative cellular reprogramming in aging and cancer.

## MATERIALS AND METHODS

### Molecular docking

The human histone demethylase LSD1 (UniProt code O60341)/REST corepressor 1 (UniProt code Q9UKL0)/histone H3 peptide (UniProt code P68431) ternary complex structure (3 Å resolution, PDB code 2X0L) was obtained from the Research Collaboratory for Structural Bioinformatics Protein Data Bank (PDB). The molecular structure of iadademstat was obtained from PubChem (PubChem_ID: 71543365). The specific edition of the LSD1 protein structure involving the removal of water, FAD, and histone H3 peptide, was made using PyMol 2.0 software (PyMOL Molecular Graphics System, v2.0 Schrödinger, LLC, at http://www.pymol.org/) without further optimization.

Molecular docking analysis of iadademstat against LSD1 was performed as previously described [84–88]. The selected protein structure was subjected to geometry optimization using the repair function of the FoldX algorithm [89]. To search for potential binding sites of iadademstat, a global molecular docking procedure was performed with AutoDock/Vina using YASARA v19.4.27 software [90, 91], where a total of 999 flexible docking runs were set and clustered (7 Å) around the putative binding sites. The YASARA pH command was set to 7.4. The docking software has a scoring function to give an approximate calculation of the Gibbs free energy variation (ΔG, kcal/mol) between LSD1 and iadademstat in each binding pose, with more positive energy values indicating stronger binding [92]. All the values were included in the corresponding table with a negative sign; only the ΔG value for the best compound docked in each cluster is shown in Table 1. To calculate this parameter, which is used to rank compounds, Autodock Vina uses a force field scoring function that considers the strength of electrostatic interactions, hydrogen bonding between all atoms of the two binding partners in the complex, intermolecular van der Waals forces, and also solvation and entropy contributions [93]. Docking results usually cluster around certain hot spot conformations. Two complexed compounds were considered to belong to different clusters if the ligand Root-Mean-Square Deviation of their atomic positions was greater than a minimum of 6 Å. Dissociation constants were recalculated from the average binding energy of all compounds of each cluster. The number of iadademstat-docked molecules included in each compound cluster is indicated as "members", as a percentage in Table 1. The key residues of LSD1 interacting with iadademstat in each cluster were detected using also YASARA v19.4.27 software [90, 91]. All of the figures were prepared using PyMol 2.0 software and all interactions were detected using the PLIP algorithm [94].

### Molecular dynamics simulations

YASARA dynamics v19.4.27 was also used for all the MD simulations with AMBER14 as a force field. The simulation cell was allowed to include 20 Å surrounding the protein and filled with water at a density of 0.997 g/mL. Initial energy minimization was carried out under relaxed constraints using steepest descent minimization. Simulations were performed in water at constant pressure-constant temperature (25°C) conditions. To mimic physiological conditions, counter ions were added to neutralize the system; Na^+^ or Cl^-^ were added in replacement of water to give a total NaCl concentration of 0.9% and pH was maintained at 7.4. Hydrogen atoms were added to the protein structure at the appropriate ionizable groups according to the calculated pKa in relation to the simulation pH (i.e., a hydrogen atom will be added if the computed pKa is higher than the pH). The pKa was computed for each residue according to the Ewald method [95]. All simulation steps were run by a preinstalled macro (md_run.mcr) within the YASARA suite. Data were collected every 100 ps.

TMM/PBSA was implemented with the YASARA macro md_analyzebindenergy.mcr to calculate the binding free energy with solvation of iadademstat, complex, and free protein for the LSD1 form complexes. The binding free energy (kcal/mol) was expressed according to the following equation:

ΔE_binding_ = [*poterec*(i) + *solverec*(i) + *potelig* + *solvelig*] - [*potecmp*(i) + *solvecmp*(i)]

where i is the position number, “pote” is the potential energy for the complex (*potecmp*), free protein (*poterec*), or free ligand (*potelig*), and “solve” is the solvation energy for the complex (*solvecmp*), free protein (*solverec*), or free ligand (*solvelig*). More positive binding free values indicate better binding.

### LSD1 enzymatic activity

Enzymatic reactions were performed in an AlphaScreen format in duplicate at room temperature for 60 minutes in a 10 μL mixture containing assay buffer, histone H3 peptide substrate, LSD1 (BPS#50103, lot#130806-D) enzyme, and iadademstat (RG-6016, Cat. No. S7795, Selleckchem.com). The 10-μL reactions were carried out in 384-well Optiplates (Perkin Elmer Life Sciences, Waltham, MA). A serial dilution of the compounds was first performed in 3.3% DMSO/assay buffer. From this step, 3 μL of iadademstat was added to 4 μL of enzyme and incubated for 30 minutes at room temperature. After this incubation, 3 μL of substrate was added to initiate the reaction. The final DMSO concentration was 1%. After the reaction, 5 μL of anti-mouse acceptor beads (Perkin Elmer, diluted 1:500 with 1× detection buffer) or 5 μL of anti-rabbit acceptor beads (Perkin Elmer, diluted 1:500 with 1× detection buffer) and 5 μL of primary antibody (BPS#52140E,F, diluted 1:200 with 1x detection buffer) were added to the reaction mix. After brief shaking, the plate was incubated for 30 minutes. Finally, 10 μL of AlphaScreen streptavidin-conjugated donor beads (Perkin Elmer, diluted 1:125 with 1× detection buffer) were added. After 30 minutes, the samples were measured in the AlphaScreen microplate reader (EnSpire Alpha 2390 Multilabel reader, Perkin Elmer).

The AlphaScreen intensity data were analyzed and compared using Graphpad Prism software (GraphPad Software Inc., San Diego, CA). In the absence of iadademstat, the AlphaScreen or fluorescence intensity (F_t_) was defined as 100% activity. In the absence of enzyme, the intensity (F_b_) was defined as 0% activity. The percent activity in the presence of iadademstat was calculated according to the following equation: %activity = (F-F_b_)/(F_t_-F_b_), where F=the A-screen intensity in the presence of iadademstat. Once A-screen data were converted to LSD1 activity (%), those values were then plotted against a series of iadademstat concentrations using non-linear regression analysis of sigmoidal dose-response curves generated with the equation Y=B+(T-B)/1+10^((LogEC50-X)×Hill Slope)^, where Y=percent activity, B=minimum percent activity, T=maximum percent activity, X=logarithm of compound and Hill Slope=slope factor or Hill coefficient. The IC_50_ value was determined as the concentration of iadademstat causing a half-maximal inhibition of control activity.

### SOX2 profiling in breast cancer datasets

We interrogated the publicly available METABRIC breast cancer dataset in the United Kingdom and Canada [40], in which mRNA expression was measured using the Illumina HT-12v13 platform and CNA with the Affymetrix SNP 6.0 array. Gene-level expression files from METABRIC were downloaded from the cBioportal for Cancer Genomics (https://www.cbioportal.org/). We used the 3-gene, PAM50, and integrative clusters subtypes provided in the METABRIC dataset.

### Breast cancer xenograft models

For generation of BRE-0188 and BRE-0192 PDX models, all clinical samples were collected under written informed consent (according to the Declaration of Helsinki) and a declaration for commercial use of the samples from the Consultative Committee for the Protection of Persons in Biomedical Research (CCPPRB) of Dijon University Hospital under authorization by a French Ministry of Higher Education, Research and Innovation for human tissue collection, and redistribution (CSP articles L 1243-3, L 1243-4, and L 1245-5). PDX tumors were passaged by serial transplantation in immunocompromised mice [50].

### Mammosphere formation

Mammosphere formation was monitored using Cell2Sphere™ assays (StemTek Therapeutics, Bilbao, Spain) as per the manufacturer’s instructions [96, 97]. Graded concentrations of iadademstat were added to triplicate sets of wells on day 1 and the number of either 6- (MDA-MB-436, BT-474) or 9-day-old (BRE-0188, BRE-0192 PDX) mammospheres was recorded as a measurement of CSC content. Images were recorded using a BioTek Cytation 5 image cytometer at 2.5× magnification. Prior to image acquisition, spheroid cultures were stained with a fluorescent vital dye to increase the accuracy of spheroid detection and analysis. The system was then set to count number, size, and aspect ratio of the objects. Thresholds were set to >100 μm in size and 0.4 as aspect ratio (with 1 being the aspect ratio of a perfect circle). Aspect ratio did not vary upon iadademstat dosage.

### Cell viability

Cell viability was determined using a standard colorimetric MTT-based reduction assay 72 h after exposure to graded concentrations of iadademstat.

### Aldefluor^®^ activity assay

The Aldefluor^®^ assay was performed as per the manufacturer’s instructions (StemCell Technologies, Vancouver, BC, Canada), with or without the addition of graded concentrations of iadademstat for 72 h. Analysis was performed using a MACSQuant^®^ Analyzer 10 flow cytometer (Miltenyi Biotec, Bergisch Gladbach, Germany) for data acquisition.

### SOX2 enhancer reporter assay

BT-474 cells were transfected with 5 μg of pGL3 Luc control (Promega, Madison, WI, USA) or pGL2-Sox2-enhancer-Luc reporter plasmids [32, 33] using Lipofectamine Plus (Invitrogen, Carslbad, CA). Twenty-four hours after transfection, the culture was split into two parts: one part was seeded in two-dimensional adherent culture plates and the other part was cultured in non-adherent culture conditions to allow mammosphere formation, in the absence or presence of graded concentrations of iadademstat. After 48 h, cells were harvested and luciferase activity was measured in duplicate with the Glomax 20/20 luminometer (Promega) and normalized by protein concentration in the extracts. Results were expressed as fold induction of sphere culture reporter activity above adherent culture control.

### Quantitative real-time PCR

Total RNA was extracted from mammosphere cells using the Qiagen RNeasy Kit according to the manufacturer’s instructions. One microgram of total RNA was reverse-transcribed to cDNA using the Reaction Ready™ First Strand cDNA Synthesis Kit (SABiosciences, Frederick, MD). PCR arrays were processed according to the SABiosciences RT-PCR manual and analyzed using an Applied Biosystems 7500 Fast Real-Time PCR System with an automated baseline and threshold cycle detection. The data were interpreted using the web-based PCR array analysis tool from SABiosciences.

### Statistical analysis

All statistical analyses were performed using GraphPad Prism software. Cell-based experimental data are presented as mean ± S.D. Comparisons of means of ≥ 3 groups were performed by analysis of variance (ANOVA) and the existence of individual differences, in case of significant *F* values at ANOVA, were assessed by multiple contrasts. The expression of SOX2 in the breast cancer subtypes (METABRIC dataset) was examined using one-way ANOVA (Kruskal-Wallis) with Dunnett’s test. *P* values < 0.05 were considered to be statistically significant (denoted as *). All statistical tests were two-sided.

## Supplementary Material

Supplementary Figure 1
