# Supplementary material for: The LSD1 inhibitor iadademstat (ORY-1001) targets SOX2-driven breast cancer stem cells: a potential epigenetic therapy in luminal-B and HER2-positive breast cancer subtypes
Source: Aging (Albany NY). 2020 Mar 18;12(6):4794–814. doi: 10.18632/aging.102887 (PMC7138538; doi:10.18632/aging.102887)
Supplement: Supplementary Figure 1 [file aging-12-102887-s001..pdf]

SUPPLEMENTARY FIGURE

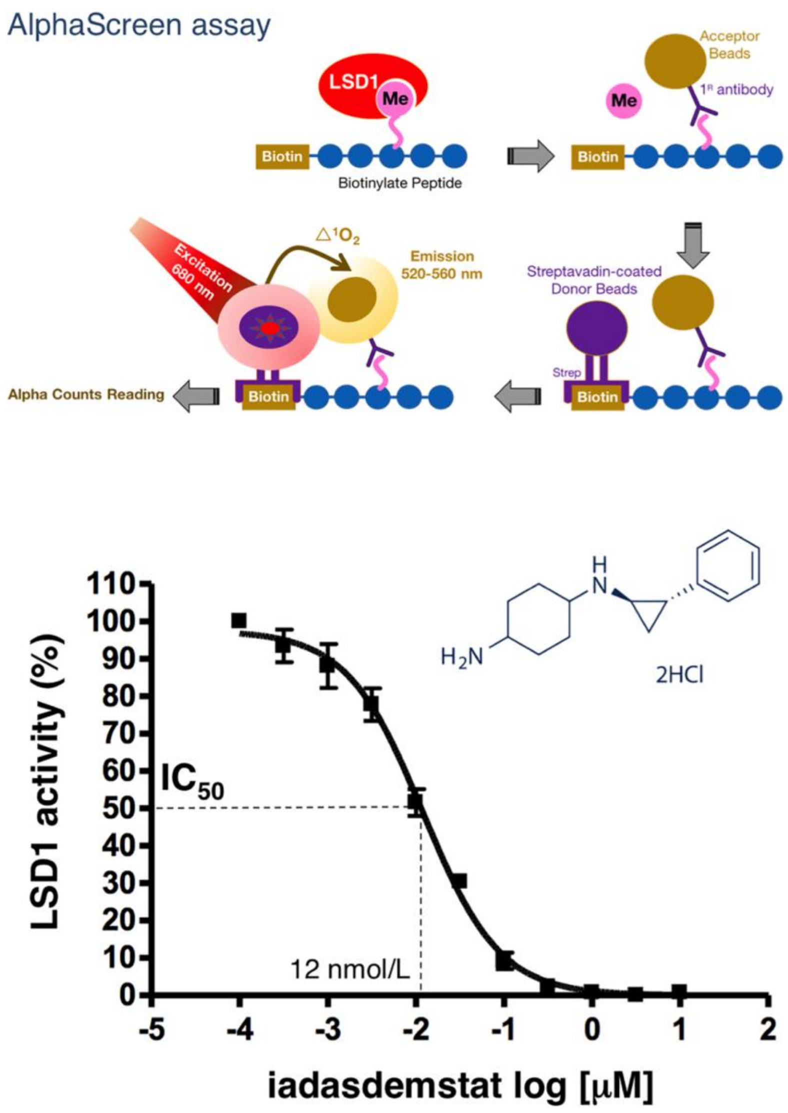

Supplementary Figure 1. Iadademstat inhibits LSD1 activity. Dose-response curves of LSD1 demethylation activity were created by plotting AlphaScreen signals as the function of iadademstat concentration. Circles and error bars represent mean values and S.D., respectively. Data are representative of two independent experiments (**P* < 0.05).
